# Identification of m6a-related signature genes in esophageal squamous cell carcinoma by machine learning method

**DOI:** 10.3389/fgene.2023.1079795

**Published:** 2023-01-17

**Authors:** Qi-Xin Shang, Wei-Li Kong, Wen-Hua Huang, Xin Xiao, Wei-Peng Hu, Yu-Shang Yang, Hanlu Zhang, Lin Yang, Yong Yuan, Long-Qi Chen

**Affiliations:** West China Hospital, Sichuan University, Chengdu, Sichuan, China

**Keywords:** M6A, RNA methylation, esophageal squamous cell carcinoma, machine learning, experimental validation

## Abstract

**Background:** We aimed to construct and validate the esophageal squamous cell carcinoma (ESCC)-related m6A regulators by means of machine leaning.

**Methods:** We used ESCC RNA-seq data of 66 pairs of ESCC from West China Hospital of Sichuan University and the transcriptome data extracted from The Cancer Genome Atlas (TCGA)-ESCA database to find out the ESCC-related m6A regulators, during which, two machine learning approaches: RF (Random Forest) and SVM (Support Vector Machine) were employed to construct the model of ESCC-related m6A regulators. Calibration curves, clinical decision curves, and clinical impact curves (CIC) were used to evaluate the predictive ability and best-effort ability of the model. Finally, western blot and immunohistochemistry staining were used to assess the expression of prognostic ESCC-related m6A regulators.

**Results:** 2 m6A regulators (YTHDF1 and HNRNPC) were found to be significantly increased in ESCC tissues after screening out through RF machine learning methods from our RNA-seq data and TCGA-ESCA database, respectively, and overlapping the results of the two clusters. A prognostic signature, consisting of YTHDF1 and HNRNPC, was constructed based on our RNA-seq data and validated on TCGA-ESCA database, which can serve as an independent prognostic predictor. Experimental validation including the western and immunohistochemistry staining were further successfully confirmed the results of bioinformatics analysis.

**Conclusion:** We constructed prognostic ESCC-related m6A regulators and validated the model in clinical ESCC cohort as well as in ESCC tissues, which provides reasonable evidence and valuable resources for prognostic stratification and the study of potential targets for ESCC.

## Highlights

1. Identified ESCC-related m6A regualtors (YTHDF1 and HNRNPC) using machine learning methods
2. Build a risk prediction model with YTHDF1 and HNRNPC
3. High expression of YTHDF1 and HNRNPC is associated with poor prognosis in ESCC patients

## 1 Introduction

Esophageal cancer (EC) ranks as the 7th malignancy worldwide in terms of its mortality rate with a 5-year survival ratio of 15%–20% (Bray et al., 2020). According to the National Central Cancer Registry of China (NCCR) statistics, Chinese EC patients accounting for 70% of all over the world 4), where esophageal squamous cell carcinoma (ESCC) takes up 88.84% of all Chinese EC patients, which is regarded as the predominant histological subtype of esophageal cancer (Zeng et al., 2016). ESCC was demonstrated to be associated with worse survival outcomes compared with other cancers (Zeng et al., 2015; Li et al., 2017). Neoadjuvant chemotherapy or chemoradiotherapy has been regarded as standard treatment for ESCC with the benefit of improving surgical outcomes (de Gouw et al., 2019), but actual clinical efficacy remains to be optimized. Owing to the specific mechanisms lied in ESCC were poorly demonstrated, meanwhile, drug resistance, and the low survival rate especially for metastatic EC patients treated with chemotherapy challenging the effectiveness of target therapies for EC, therefore, taking the pathogenesis of ESCC as a starting point to find potential diagnostic and therapeutic targets for ESCC is the current research direction on ESCC (Abnet et al., 2018). Previous studies have reported that abnormal N6-methyladenosine (m6A) methylation levels play an important role in the progression of various cancers (Chen and Wong, 2020). However, the research of m6A in ESCC is still in its infancy, therefore, from the perspective of m6A methylation modification, the identification of new and reliable prognostic predictors is very important for developing appropriate ESCC treatment strategies and improving the poor prognosis of ESCC patients.

Numerous studies in recent years have shown RNA methylation is a common seen in human and it is also a key regulator of transcriptional expression. N6-methyladenosine (m6A), occurs in approximately 25% of genome-wide transcripts and occurs around stop codons, 5′- and 3′-untranslated regions, and long intra-exon enrichment, is regarded as one of the most frequent RNA methylation (Dominissini et al., 2012; Meyer et al., 2012). RNA m6A modifications regulate RNA splicing, translocation, stability, and protein translation, catalyzed by methyltransferase complexes, including methyltransferase-like 3 and 14 proteins (METTL3 and METTL14) and their co-factors: WTAP, RBM15, RBM15B, and ZC3H13 (Lan et al., 2019). The demethylation is mediated by “eraser” proteins (ALKBH5 and FTO) (Huang et al., 2019; Jin et al., 2020a). Finally, m6A modifications show the biological functions by binding to m6A “readers,” including YTH domain-containing proteins (YTHDC1-2), YTH-family proteins (YTHDF1-3), insulin-like growth factor 2 mRNA-binding proteins (IGF2BP1-3), HNRNPA2B1, HNRNPC and RBMX (Huang et al., 2018a; Jiang et al., 2021). Meanwhile, aberrant global m6A abundance also provides more possibilities for the early diagnosis and treatment of various cancers, such as bladder cancer (Chen et al., 2019), liver cancer (Chen and Wong, 2020), lung cancer (Zhang et al., 2020a), gastrointestinal cancer (Sun et al., 2019), and invasive malignant pleomorphic adenoma with different methylation levels (Han H et al., 2021). In ESCC, Mettl3 promotes esophageal cancer initiation and progression (Chen X et al., 2021; Han Z et al., 2021b; Wang et al., 2021), followed by ALKBH5 (Nagaki et al., 2020; Xu et al., 2020; Xiao et al., 2021), FTO (Liu et al., 2020), YTHDC2 (Hu et al., 2020), HNRNPA2B1 (Guo et al., 2020) and HNRNPC (Xu et al., 2020). These m6A regulators have all been studied to regulate ESCC progression through m6A modification of downstream target genes. However, most studies combined bioinformatic analysis of open access databases to obtain m6A RNA methylation regulators that may play an important role in ESCA carcinogenesis, but lacked validation of large-scale clinical sample whole-genome sequencing data. Thus, the regulators of m6A methylation acting on ESCC are diverse and differ from study to study.

Nowadays, precision medicine has become more and more important in the diagnosis and treatment of diseases. However, with the massive expansion of available genomic data, and novel disease biomarkers, new analytical challenges arise to decipher complex relationships between vast amounts of information and multiple data types. Machine learning can detect indistinguishable patterns from large, noisy or complex datasets. This capability is particularly suitable for complex genomic data, especially in cancer research (Simes, 1985; Cicchetti, 1992). At present, machine learning is mostly used in Cancer Classification and Subtyping, Biomarker/Signature Discovery, Drug Discovery for Cancer Therapy, Cancer Driver Gene Discovery and Cancer Gene/Protein Interaction and Networks (Huang et al., 2018b). The prediction of m6A modification sites in disease is also found in glioma (Du et al., 2021) Soft Tissue Sarcoma (Huang et al., 2021a), gastric cancer (Zhang et al., 2020b), hepatocelluar carcinoma (Huang et al., 2021b) etc. However, in ESCC, no machine learning algorithm has been applied to find out ESCC related m6a regulators.

In this study, we identified differentially expressed m6A RNA methylation regulators between normal and tumor samples using whole-genome transcriptome sequencing data from 66 patients diagnosed with esophageal squamous cell carcinoma at our hospital. Modeling was performed using machine learning methods, the prediction effect of the model was evaluated, and the most important m6A genes that could distinguish ESCC from normal groups were screened. RNA-sequencing data from The Cancer Genome Atlas (TCGA)-ESCA dataset was then screened for differentially expressed m6A RNA methylation regulators between normal and tumor samples using the same machine learning and modeling approach. After taking the intersection between the two data sets, a risk prediction model is established. Finally, our results were validated in tissue samples and an independent clinical ESCC cohort.

## 2 Meterials and methods

### 2.1 Patients and datasets collection

We used the RNA-seq data of 66 pairs of ESCC samples collected from the Department of Thoracic Surgery, West China Hospital of Sichuan University between 2020 and 2021 as the training cohort, which contained complete clinical characteristic data, including gender, age, tumor differentiation, TNM stage, and surgical method. In addition, we downloaded expression profiles of 159 ESCC samples from the TCGA-ESCA database (https://portal.gdc.cancer.gov/) as the validation cohort. Normalization of transcriptome counts was performed by the edgeR package (version 3.26.8).

In terms of experimental validation, a total of 5 pairs of ESCC tissues and adjacent normal tissues (8–10 cm from the original tumor boundary) were collected from patients who underwent radical esophagectomy at our hospital from June 2022 to July 2022 were. All patients were informed of the risks of the operation. Permission to use resected specimens and written consent were obtained from the study participants preoperatively.

### 2.2 Machine learning methods

We employed two machine learning approaches: RF (Random Forest) and SVM (Support Vector Machine) to screen ESCC-related m6A regulators. The RF modeling adopts the “Repeatedcv” method in the R language train function, and the SVM modeling adopts the “svmRadial” method. The outcome variables were normal group and ESCC group, and differential genes were put into the model as independent variables. Plot the boxplots and cumulative residual distributions of the two models to determine which model performs better. If the gene importance score is greater than 2, it is regarded as a characteristic gene (Watson et al., 2019).

A nomogram was constructed using the eigengenes screened by machine learning to visualize the predictive power of the eigengenes for the control group and the ESCC group. The nomogram formulates the scoring standard according to the size of the regression coefficients of all independent variables, and assigns a score to each independent variable; for each patient, a total score can be calculated, and then through the conversion between the score and the probability of the outcome, probability of each patient’s outcome happening can be calculated. Calibration curves, clinical decision curves, and clinical impact curves (CIC) were used to evaluate the predictive ability and best-effort ability of the model. Statistics and graphics were performed using R-Studio (version 4.2.1). Two-sided *p* < .05 was considered statistically significant.

### 2.3 Western blotting

After washing with cold phosphate-buffered saline (PBS) and pelleting, the protein concentration was determined using a bicinchoninic acid assay. After electrophoresis on SDS-PAGE, proteins were transferred onto PVDF membranes. The membranes were blocked with 5% nonfat milk and incubated with primary antibodies at 4°C overnight. The corresponding horseradish peroxidase (HRP)-conjugated secondary antibody was added and incubated at room temperature for 2 h. Signals were visualized using an enhanced chemiluminescence reaction with an HRP substrate. The primary antibodies against YTHDF1 (1:150, A18126) and HNRNPC (1:200, A19137) were purchased from Abclonal, China. The antibody against *β*-actin was purchased from Sigma-Aldrich Co. (St Louis, MO, United States).

### 2.4 Immunohistochemistry staining and scoring

The ESCC tissue sections included 120 cases of ESCC from February 2016 to June 2017 with complete clinical information were collected and produced by our team. Immunohistochemical staining was performed subsequently. The tissue sections were first kept at 60°C for 24 h. Xylene deparaffinization and hydration were then carried out with an ethanol gradient (100%–60%). Antigen retrieval was performed by heating sections in 10 mM citrate (pH 6.0) boiling buffer for 15 min. ESCC tissue sections were incubated overnight at 4°C with rabbit monoclonal anti-YTHDF1 antibody (1:1,000, A18126, Abclonal, China) and anti-HNRNPC (1:2000, A19137, Abclonal, China). Incubation with the corresponding secondary antibody (Rabbit IgG, 1:5,000, Santa Cruz Biotechnology) was performed the next day at room temperature (25°C) for 30 min, followed by staining with 3, 3′-diaminobenzidine (DAB) and hematoxylin. The results of ESCC tissue sections were viewed and photographed with the Olympus BX53 fluorescence microscope (Tokyo, Japan). A composite score was determined using a previously described method (Laffitte et al., 2001).

### 2.5 Statistical analysis

The clinicopathological characteristics of the patients were analyzed by Pearson’s chi-square test or Fisher’s exact test to compare the dichotomous variables. Student’s t-test was applied for the mean values of continuous variables that conformed to a normal distribution; the others were analyzed using the Mann-Whitney *U* test. Overall survival (OS) in patients was analyzed using Kaplan-Meier curves, and log-rank tests were used to determine the statistical significance. Multivariate survival analysis was carried out with the Cox proportional hazard regression model, in which the covariates that met the proportional hazards assumption of the covariate interaction test were considered to pass. All statistical tests were two-sided. A *p*-value of less than .05 was considered statistically significant.

## 3 Results

### 3.1 Expression of m6A RNA methylation regulators in esophageal squamous cell carcinoma

**FIGURE 1:**
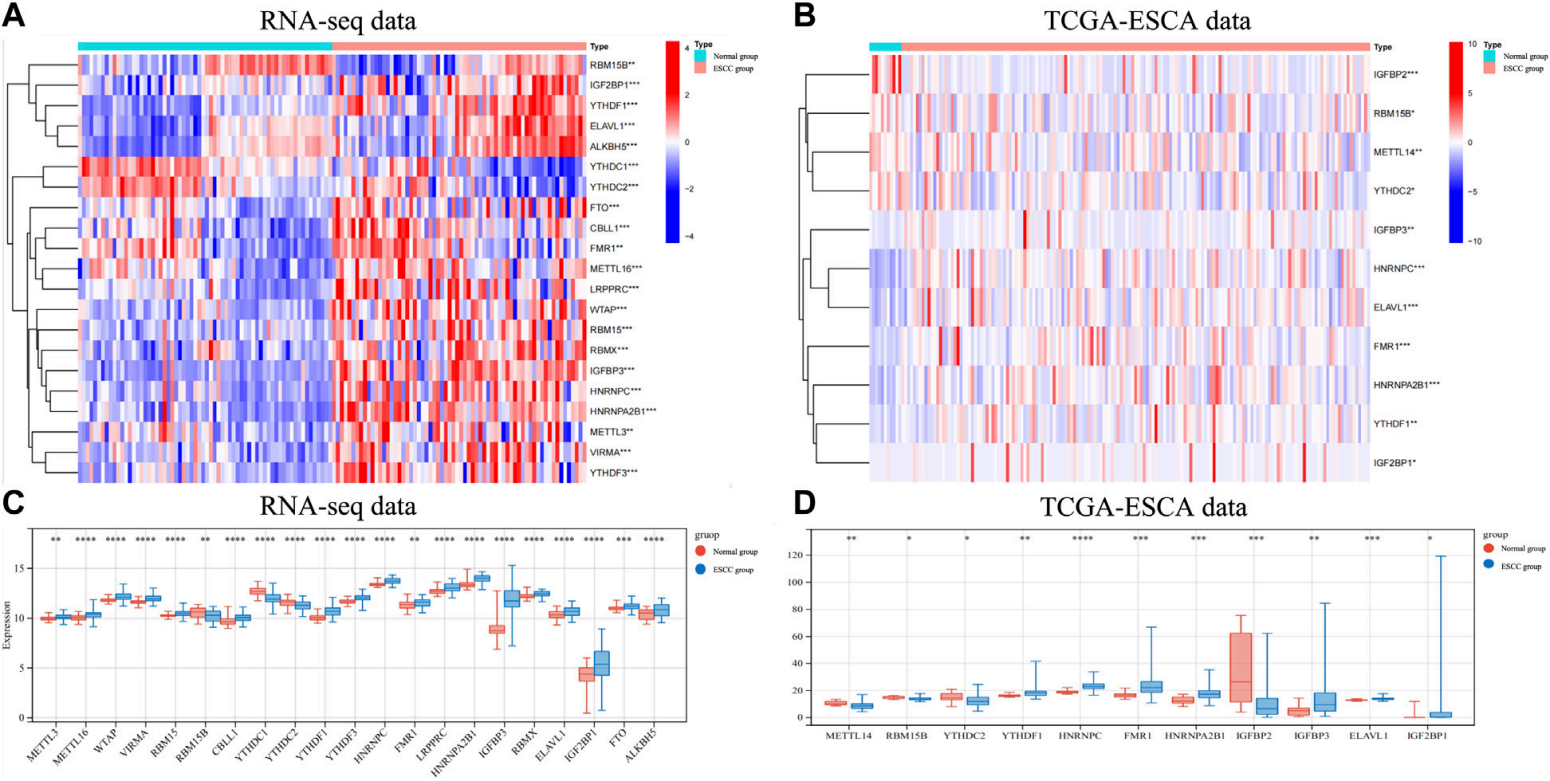
The expression of different m6A RNA methylation regulators in RNA-seq data andin TCGA-ESCA data. **(A)** The heatmap showed 21 significantly different expression of m6A regulators in each sample extracted from our RNA-seq data. **(B)** The heatmap of 11 differentially expressed m6A regulators in each sample extracted from TCGA-ESCA database. **(C)** The expression of 21 differentially expressed m6A regulators between normal and ESCCtissues in our RNA-seq cohort. **(D)** The expression of 11 differentially expressed m6A regulators between normal and ESCC tissues in TCGA-ESCA cohort. Data are shown as the means ± SD. *p < 0.05, **p < 0.01, ***p < 0.001; m6A, N6-methyladenosine; ESCA, esophageal squamous cell carcinoma; TCGA, The Cancer Genome Atlas.

We used the transcriptome data of 66 ESCC samples collected from the Department of Thoracic Surgery, West China Hospital of Sichuan University to analyze the mRNA expression levels of m6A RNA methylation regulators. A heatmap was generated to visualize the expression of 21 m6A regulators that were significantly different between ESCC and normal tissues (Figure 1A). The mRNA expression levels of 18 m6A regulators (METTL3, METTL16, WTAP, VIRMA, RBM15, CBLL1, YTHDF1, YTHDF3, HNRNPC, FMR1, LRPPRC, HNRNPA2B1, IGFBP3, RBMX1, ELAV1, IGF2BP1, FTO, and ALKBH5) were significantly increased in ESCC compared with normal tissues. The other 3 regulators (RBM15B, YTHDC1, YTHDC2) were downregulated in ESCC (Figure 1B). We then used the same approach to analyze the mRNA expression levels of m6A RNA methylation regulators using transcriptome data from the TCGA-ESCA database. Similarly, we found that the mRNA expression of 7 m6A regulators (YTHDF1, HNRNPC, FMR1, HNRNPA2B1, IGFBP3, ELAVL1, and IGF2BP1) was significantly upregulated in ESCC compared to normal tissues, while METTL3, RBM15B, YTHDC2, and IGFBP2 were significantly downregulated in ESCC.

### 3.2 Screening esophageal squamous cell carcinoma-related m6A regulators using machine learning

**FIGURE 2:**
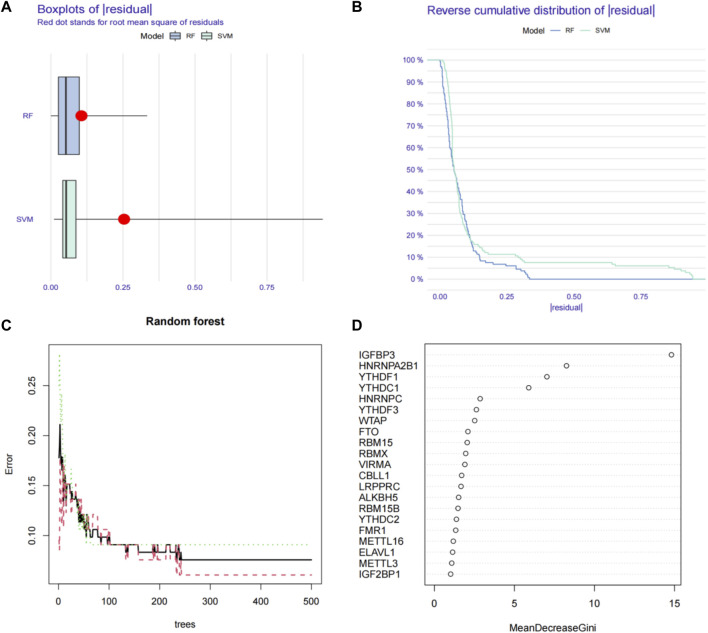
Using machine learning to find ESCC-related m6A regulators in our RNA-seq data. **(A)** Boxplots of residual values of RF (Random Forest) and SVM (Support Vector Machine) models. Red dot represents the mean residual value. **(B)** Cumulative residual distribution plots of RF (Random Forest) and SVM (Support Vector Machine) models. **(C)** The error value of the random forest. The red line represents the error of the ESCC group, the green line represents the error of the Normal group, and the black line represents the total sample error. The minimum error point of the three-group cross-validation is 0.076, corresponding to 233 optimal random forest trees. **(D)** Mean Decrease Gini score of 21 differentially expressed m6A regulators extracted from RNA-seq data. RF, Random Forest; SVM, Support Vector Machine; m6A, N6-methyladenosine.

**FIGURE 3:**
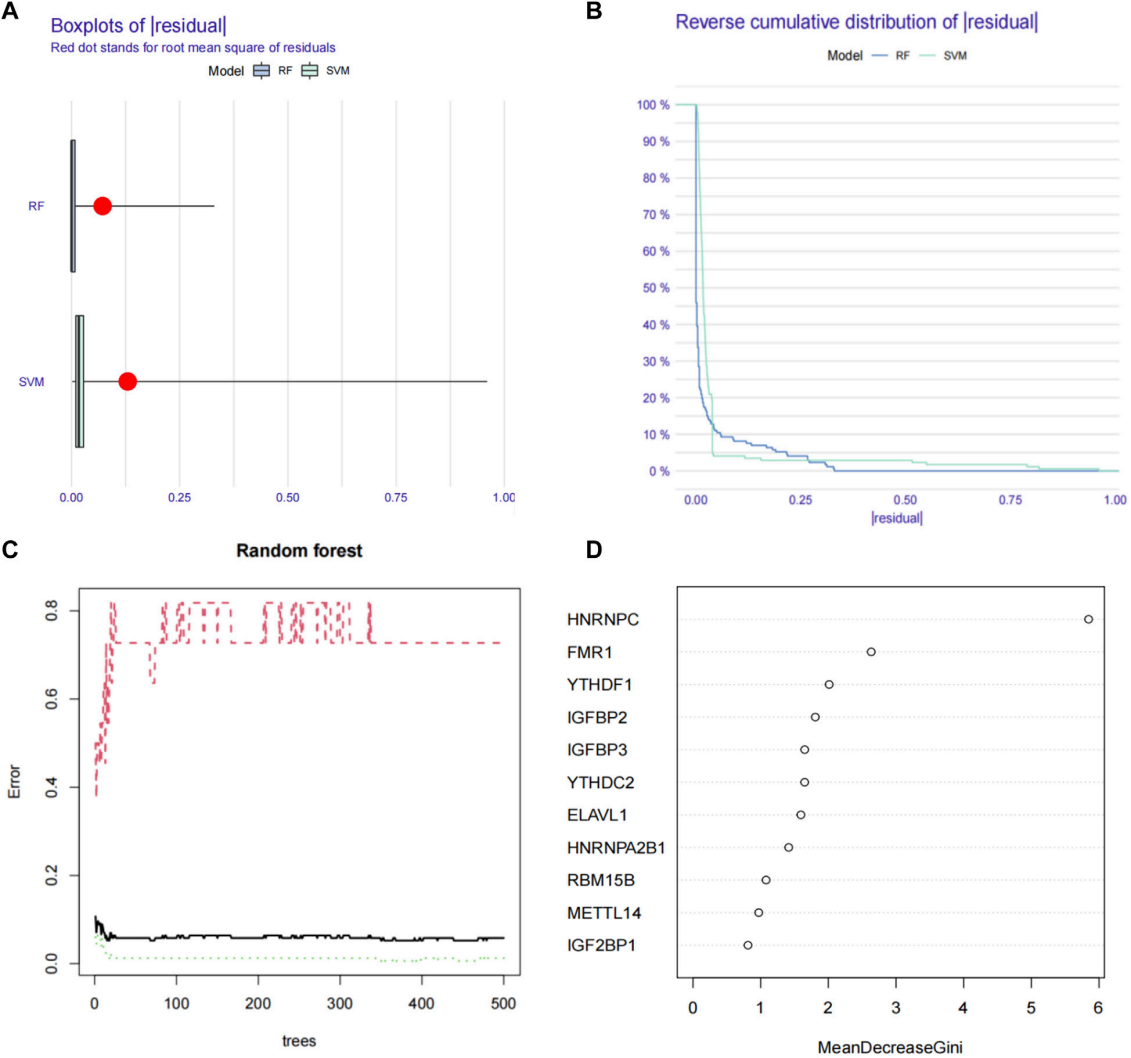
Using machine learning to find ESCC-related m6A regulators in TCGA-ESCA database. **(A)** Boxplots of residual values of RF (Random Forest) and SVM (Support Vector Machine) models. Red dot represents the mean residual value. **(B)** Cumulative residual distribution plots of RF (Random Forest) and SVM (Support Vector Machine) models. **(C)** The error value of the random forest. The red line represents the error of the ESCC group, the green line represents the error of the Normal group, and the black line represents the total sample error. The minimum error point of the three-group cross-validation is is 0.076, corresponding to 18 optimal random forest trees. **(D)** Mean Decrease Gini score of 11 differentially expressed m6A regulators extracted from TCGA-ESCA database. RF, Random Forest; SVM; Support Vector Machine; m6A, N6-methyladenosine; TCGA, The Cancer Genome Atlas.

We first used the RF (random forest) and SVM (support vector machine) machine learning methods on the transcriptome data of our hospital to take the expression of the above 21 m6A genes as independent variables, and the normal group and ESCC group as the outcome variables into RF and SVM models. We analyzed the boxplots and cumulative residual distributions of both models to determine which model had better performance. Figure 2A shows that the mean residual value for RF is .00467 and SVM is .00471. The residual inverse cumulative distribution line of RF lies mostly within the residual line of SVM (Figure 2B), indicating that the difference between the predicted value of RF and the true value is smaller, and the model is more accurate. Therefore, we chose the RF model to predict ESCC-related m6A genes. As can be seen from Figure 2C, the cross-validation minimum error point for ESCC group error (red), normal group error (green) and total sample error (black) is .076, corresponding to 233 optimal random forest trees. The importance score of ESCC-related m6A genes was further obtained by RF model. The higher the meanDecreaseGini score, the more important the gene is. Finally, we obtained that IGFBP3, HNRNPA2B1, YTHDF1, YTHDC1, HNRNPC, YTHDF3, WTAP, FTO, and RBM15 were m6A regulators significantly associated with ESCC (Figure 2D). Similarly, we imported the above 11 m6A regulators screened from the TCGA-ESCA database into the RF and SVM models, where the mean residual value of RF was .00122 and SVM was .00198 (Figure 3A). The residual inverse cumulative distribution line of RF lies mostly within the residual line of SVM (Figure 3B), and we chose the RF model to predict the eigengenes of ESCC. The cross-validation minimum error point of ESCC group error (red), normal group error (green) and total sample error (black) is .076, corresponding to 18 optimal random forest trees (Figure 3C). Through meanDecreaseGini score, HNRNPC, FMR1, as well as YTHDF1 were m6A regulators significantly associated with ESCC (Figure 3D).

### 3.3 Validation of esophageal squamous cell carcinoma-related m6A regulators predictive proficiency

**FIGURE 4:**
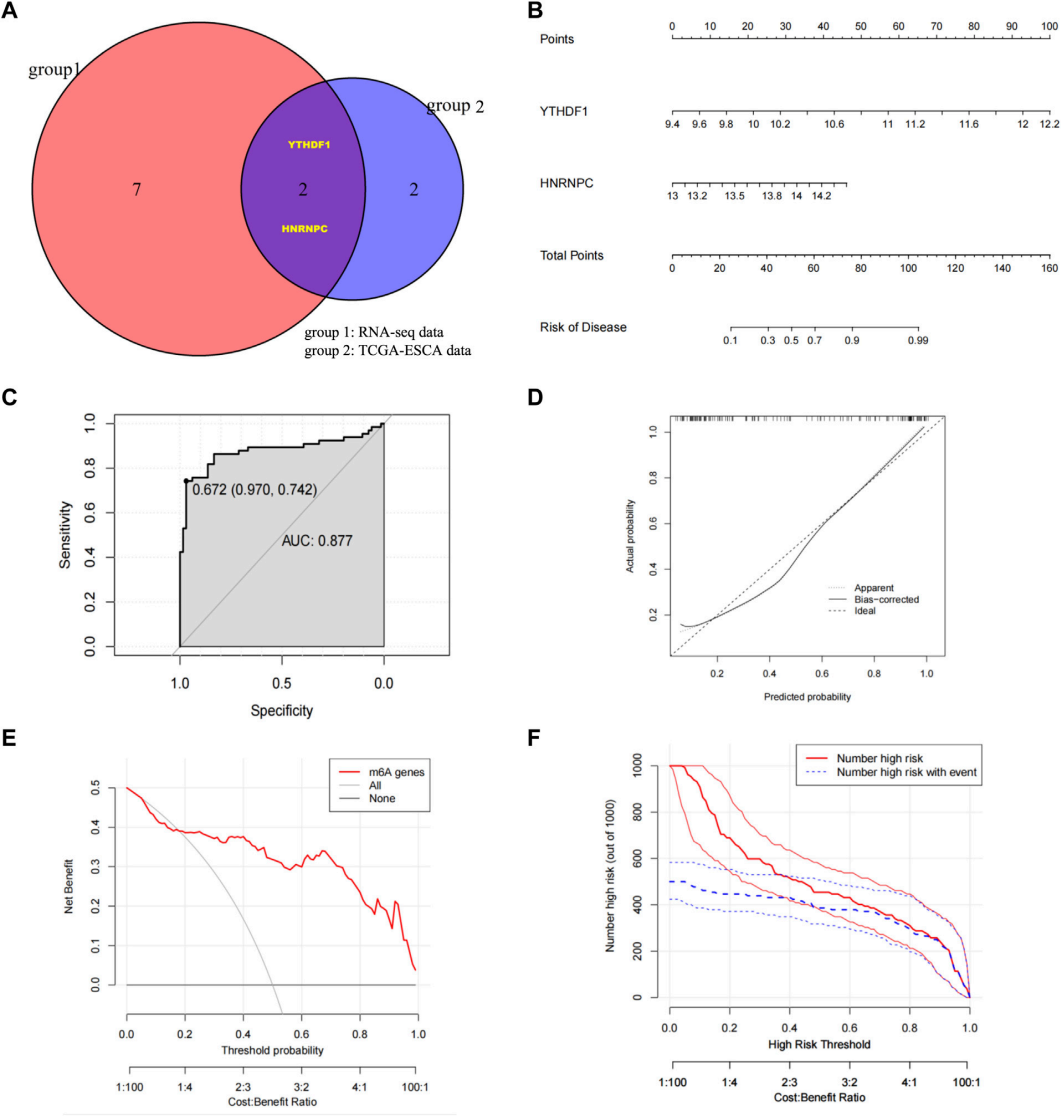
Identify ESCC-related m6A regulators, build risk models, and validate model predictive capabilities **(A)** Venn diagrams determined YTHDF1 and HNRNPC as ESCC risk factors after overlapping m6A regulators with higher meanDecreaseGini score in both RNA-seq data and TCGA-ESCA database. **(B)** Nomogram was constructed using YTHDF1 and HNRNPC to visualize the predictive ability of ESCC-related m6A regulators for Normal group and ESCC group. **(C)** Calibration curve with nomogram predicted probabilities on the x-axis and actual probabilities on the y-axis. The dashed line represents the true value, and the solid line is bias-corrected by Bootstrapping (1000 repetitions). **(D)** ROC curve was applied to assess the predictive efficiency of the model. **(E)** Clinical decision curve. The red line represents the outcome predicted by the model, the grey line represents “all positive diagnosis (ESCC)” and the black line represents “all negative diagnosis (Normal)”. The horizontal axis is Threshold Probability, and the vertical axis is the net profit rate after subtracting the disadvantages. **(F)** Clinical Impact Curve (CIC). The red curve (Number of high risk) represents the number of people classified as ESCC by the model at each threshold probability; the blue curve (Number high risk with outcome) represents the number of true ESCC at each threshold probability. AUC, Area Under Curve; CIC, Clinical Impact Curves; ESCC/ESCA, Esophageal quamous cell carcinoma; HNRNPC, RNA-binding protein, is a member of the heterogeneous ribonucleoproteins C; m6A, N6-methyladenosine; TCGA, The Cancer Genome Atlas; YTHDF1, YTH N6-methyladenosine RNA-binding protein 1.

Through Venn diagrams, the m6A regulators significantly related to ESCC screened in our RNA-seq data and TCGA-ESCA database based on machine learning were overlapped, and then the final ESCC related m6A regulators in this study were screened: YTHDF1 and HNRNPC (Figure 4A). We constructed nomograms to visually demonstrate the predictive power of ESCC-related m6A regulators for normal and ESCC groups (Figure 4B). The nomogram showed that YTHFD1 made the largest contribution to risk of ESCC, followed by HNRNPC. The coefficients are used to assign the score of these independent factors. Finally, the sum of these scores can be used to predict the risk of ESCC (Figure 4B). From the ROC curve we obtained the AUC of the model was .877 (Figure 4C). A calibration plot of the nomogram is shown in Figure 4D, which shows that the predicted risk of ESCC probability agrees well with real-world observations. At the same time, we also established a clinical decision curve prediction model. The results showed that patients had a higher net gain when YTHDF1 and HNRNPC were used as signature genes to predict the occurrence of ESCC, suggesting that the model is worth using (Figure 4E). Finally, based on the clinical decision curve, we further draw the clinical impact curve (CIC). The red curve (numeric high risk) represents the number of people classified as positive (high risk) by the model at each threshold probability. The blue curve (high-risk numbers with outcomes) is the number of true positives at each threshold probability. The results show that the blue curve is within the red curve, indicating that the model has excellent classification ability. This also suggests that YTHDF1 and HNRNPC can be considered as biomarkers significantly associated with the pathogenesis of ESCC. At the same time, we also performed the PPI analysis based on our RNA-seq data in terms of YTHDF1 and HNRNPC, and we found ⅩⅩⅩ may potentially interact with HNRNPC and ⅩⅩⅩ may potentially interact with YTHDF1, however, the specific mechanism of interaction still need further study.

### 3.4 Prognostic and clinicopathological significance of risk grouping in esophageal squamous cell carcinoma

**FIGURE 5:**
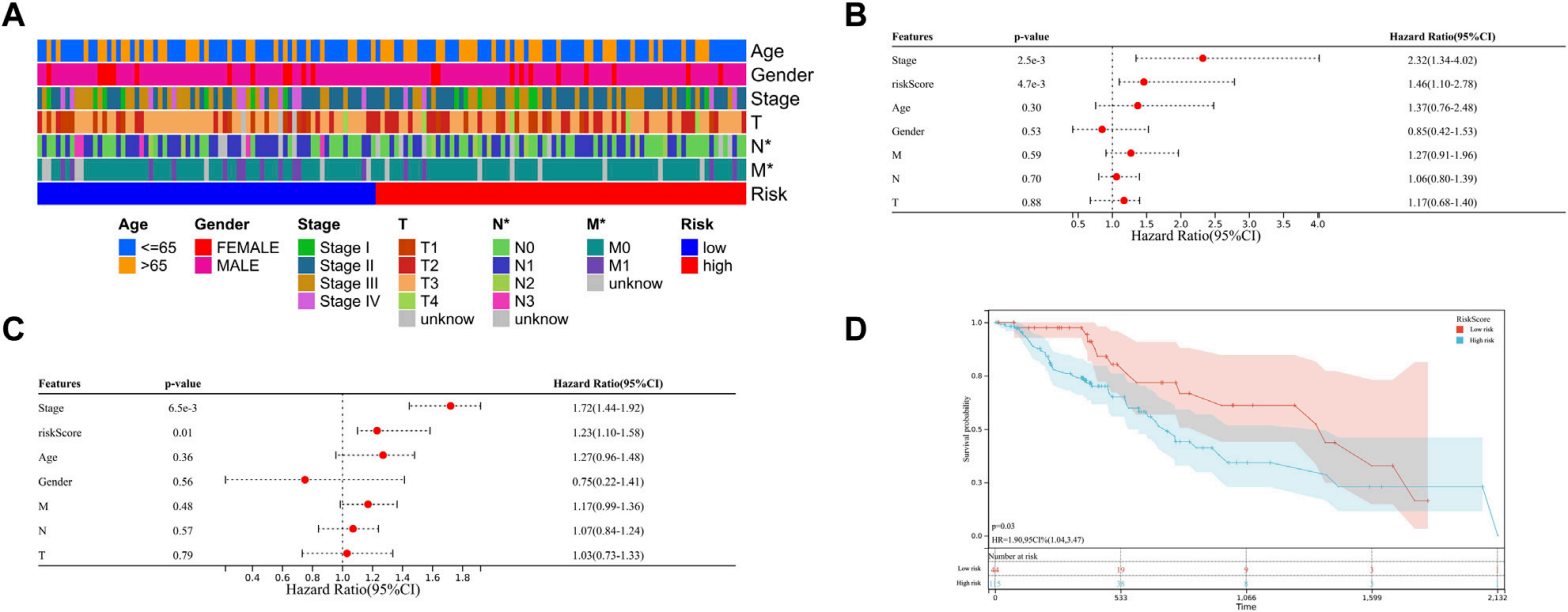
Clinical significance of risk grouping in TCGA-ESCA database **(A)** The heatmap shows the clinicopathological features were compared between the high- and low-risk groups. **(B)** Univariate Cox analysis of the risk score and clinicopathological features. **(C)** Multivariate Cox analysis of the risk score and clinicopathological features to identify the independent prognostic predictors in TCGA-ESCA database. **(D)** Kaplan-Meier analysis for patients of TCGA-ESCA database in high- and low-risk group. Data are shown as the means ± SD. *p < 0.05. ESCA, esophageal squamous cell carcinoma; TCGA, The Cancer Genome Atlas; HNRNPC, RNA-binding protein, is a member of the heterogeneous ribonucleoproteins C; YTHDF1, YTH N6-methyladenosine RNA-binding protein 1.

Regarding the AUC value was .877, the heatmap showing the difference in terms of clinicopathological features, HNRNPC, and YTHDF1 in mRNA expression levels in high- and low-risk groups was generated in TCGA-ESCA database. Notably, the high-risk group was significantly associated with advanced N stage (*p* < .05) and M stage (*p* < .05) (Figure 5A). Meanwhile, the univariate and multivariate COX analysis were conducted, in which risk grouping as well as TNM stage were significantly correlated with OS during univariate analysis in TCGA-ESCA database. When put the statistic significant signatures into the multivariate analysis, finally, risk grouping and TNM stage were identified as the independent prognostic factor of ESCC patients as well (Figures 5B,C). We then validate the prognostic role of risk grouping through Kaplan-Meier survival analysis in TCGA-ESCA cohort, which showed the patients in high-risk group got the significant worse overall survival than those in low-risk group (Figure 5D).

### 3.5 Experimental validation of YTHDF1 and HNRNPC in esophageal squamous cell carcinoma tissues and a clinical esophageal squamous cell carcinoma cohort

**FIGURE 6:**
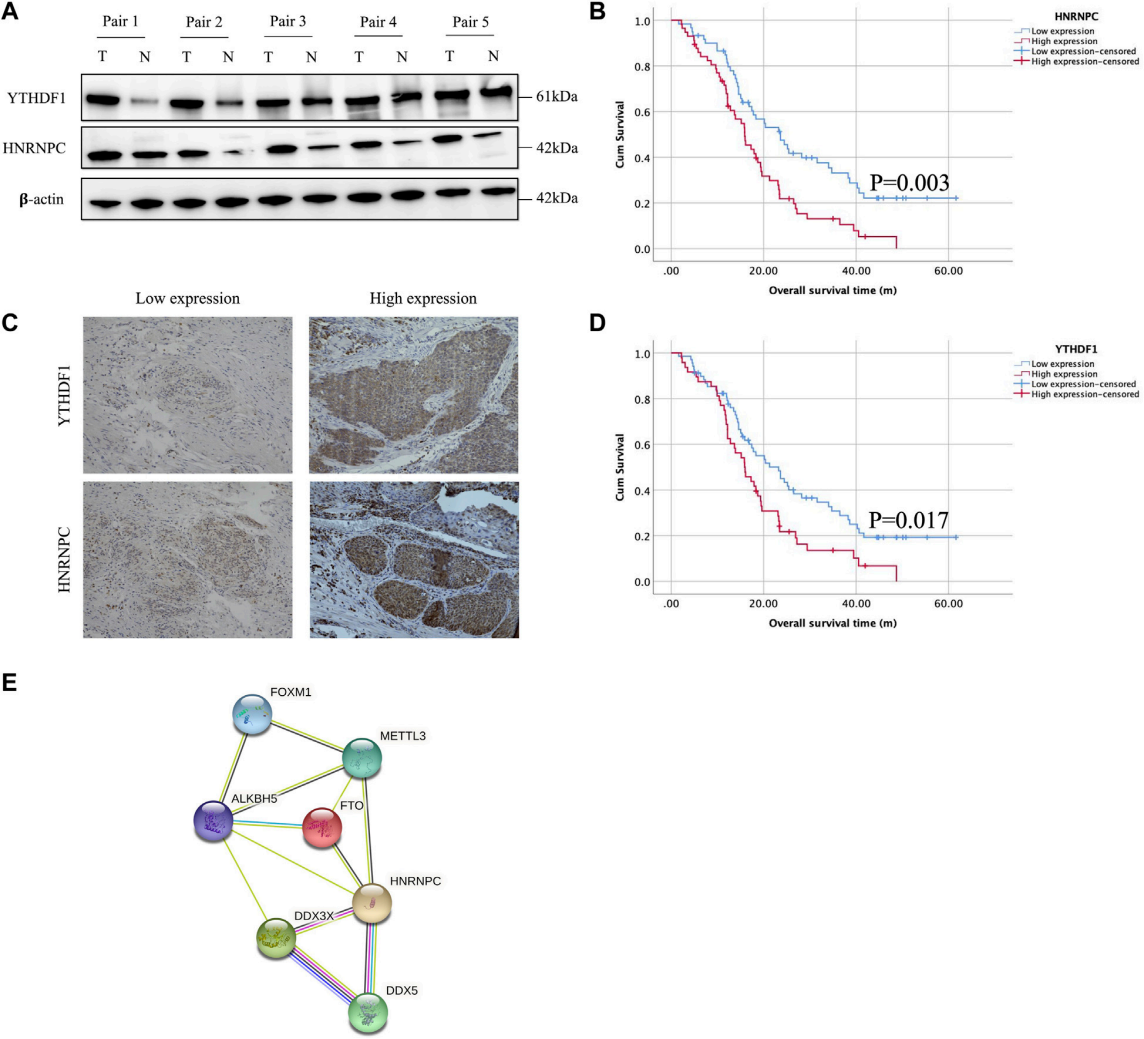
Experimental validation of YTHDF1 and HNRNPC with Western blot, immunohistochemical staining and PPI network analysis. **(A)** Western blot analysis of YTHDF1 and HNRNPC expression in 5 pairs of fresh frozen ESCC specimens with related adjacent normal tissues. **(B)** Representative immunohistochemical images of YTHDF1 and HNRNPC expression in ESCC tissues (scale bar: 50 μm). **(C)** Kaplan-Meier analysis for patients with high or low expression levels of YTHDF1 and HNRNPC from ESCC tissue sections. **(D)** PPI network showed the interactions between HNRNPC and other m6A RNA methylation regulators. Data are shown as the means ± SD. *p < 0.05. ESCA, esophageal squamous cell carcinoma; TCGA, The Cancer Genome Atlas; HNRNPC, RNA-binding protein, is a member of the heterogeneous ribonucleoproteins C; YTHDF1, YTH N6-methyladenosine RNA-binding protein 1; METTL3, Methyltransferase-like 3.

Owing to the expression of YTHDF1 and HNRNPC in mRNA level have been showed in Figure 1, in which, the results from both our RNA-seq data and TCGA-ESCA database showed the expression of YTHDF1 and HNRNPC were significantly higher than that of normal tissue samples. Then, the protein level of YTHDF1 and HNRNPC in ESCC tissue were validated in 5 pairs of fresh frozen ESCC specimens with related adjacent normal tissues collected from West China Hospital by western blot. Compared with adjacent normal tissues, the protein level of both YTHDF1 and HNRNPC in ESCC tissue were significantly higher (Figure 6A). To examine the expression of YTHDF1 and HNRNPC in ESCC more precisely, we performed immunohistochemistry on ESCC tissue sections (Figure 6B). The clinicopathological characteristics of YTHDF1 and HNRNPC expression in ESCC tissue sections are listed in Table 1 and Table 2. In terms of survival, the median follow-up time was 20.6 months, which ranged from 1.0 to 61.6 months in our study. According to the Kaplan-Meier curves, the prognosis of ESCC patients with higher HNRNPC (*p* = .003, Figure 6C) and YTHDF1 expression (*p* = .017, Figure 6D) was significantly worse, respectively. After Cox multivariate regression analysis, YTHDF1 and HNRNPC expression were shown to be the independent prognostic factor related to ESCC, respectively (Table 3). The protein–protein interactions (PPI) were analyzed *via* our RNA-seq data in order to find out the potential correlated genes with YTHDF1 or HNRNPC (Figure 6E). Correlation analysis demonstrated that all the regulators were positively correlated with each other, among which, METTL3, DDX5, DDX3X, ALKBH5 and FTO were all significantly associated with HNRNPC.

**TABLE 1:** Clinicopathological characteristics of YTHDF1 expression on ESCC tissue sections.

		YTHDF1 low expression	YTHDF1 high expression	*P*
		64 (%)	56 (%)	
Gender	Male	47 (55.3)	38 (44.7)	.319
	Female	17 (48.6)	18 (51.4)	
Age	<55	12 (48.0)	13 (52.0)	.353
	≥55	52 (54.7)	43 (45.3)	
Tumor location	Upper thoracic	4 (57.1)	3 (42.9)	.574
	Middle thoracic	48 (55.8)	38 (44.2)	
	Lower Thoracic	12 (44.4)	15 (55.6)	
T stage	T1	4 (66.7)	2 (33.3)	.004*
	T2	20 (83.3)	4 (16.7)	
	T3	40 (45.5)	48 (54.5)	
	T4	0 (.0)	2 (100.0)	
N stage	N0	38 (64.4)	21 (35.6)	.054
	N1	17 (48.6)	18 (51.4)	
	N2	6 (30.0)	14 (70.0)	
	N3	3 (50.0)	3 (50.0)	
TNM stage	I	5 (71.4)	2 (28.6)	.006*
	II	37 (68.5)	17 (31.5)	
	III	20 (36.4)	35 (63.6)	
	IV	2 (50.0)	2 (50.0)	
Differentiation	High	9 (69.2)	4 (30.8)	.114
	Moderate	52 (54.2)	44 (45.8)	
	Low	3 (27.3)	8 (72.7)	

ESCC, esophageal squamous cell carcinoma; YTHDF1: YTH N6-methyladenosine RNA-binding protein 1; * Meaningful *p*-value.

**TABLE 2:** Clinicopathological characteristics of HNRNPC expression on ESCC tissue sections.

		HNRNPC low expression	HNRNPC high expression	*P*
		62 (%)	58 (%)	
Gender	Male	42 (49.4)	43 (50.6)	.285
	Female	20 (57.1)	15 (42.9)	
Age	<55	11 (44.0)	14 (56.0)	.262
	≥55	51 (53.7)	44 (46.3)	
Tumor location	Upper thoracic	4 (57.1)	3 (42.9)	.431
	Middle thoracic	47 (54.7)	39 (45.3)	
	Lower Thoracic	11 (40.7)	16 (59.3)	
T stage	T1	3 (50.0)	3 (50.0)	<.001*
	T2	22 (91.7)	2 (8.3)	
	T3	37 (42.0)	51 (58.0)	
	T4	0 (.0)	2 (100.0)	
N stage	N0	23 (39.0)	36 (61.0)	.041*
	N1	22 (62.9)	13 (37.1)	
	N2	14 (70.0)	6 (30.0)	
	N3	3 (50.0)	3 (50.0)	
TNM stage	I	6 (85.7)	1 (14.3)	<.001*
	II	40 (74.1)	14 (25.9)	
	III	15 (27.3)	40 (72.7)	
	IV	1 (25.0)	3 (75.0)	
Differentiation	High	7 (53.8)	6 (46.2)	.566
	Moderate	51 (53.1)	45 (46.9)	
	Low	4 (36.4)	7 (63.6)	

ESCC, esophageal squamous cell carcinoma; HNRNPC: heterogeneous nuclear ribonucleoprotein C; * Meaningful *p*-value.

**TABLE 3:** Univariate and multivariate Cox regression analyses of clinical factors associated with 5-year overall survival on ESCC tissue sections.

	Univariate cox regression analysis			Multivariate cox regression analysis			Multivariate cox regression analysis		
	HR	95%CI	*P*	HR	95% CI	*P*	HR	95% CI	*P*
Gender	.823	.358, 1.892	.646						
Age	1.890	.665, 5.371	.232						
Tumor location	1.247	.745, 1.683	.445						
T stage	1.933	1.136, 3.288	.015*	1.799	1.048, 3.085	.033*	1.813	1.063, 3.091	.029*
N stage	1.722	1.219, 2.434	.002*	1.605	1.125, 2.290	.009*	1.693	1.189, 2.413	.004*
TNM stage	1.716	1.290, 2.281	<.001*	1.615	1.088, 2.398	.017*	1.589	1.093, 2.310	.015*
Differentiation	1.459	.710, 2.997	.303						
YTHDF1 expression	1.630	1.158, 2.296	.019*	1.549	1.098, 2.186	.013*			
HNRNPC expression	1.921	1.453, 5.872	.003*				1.563	1.311, 5.349	.007

95% CI, 95% Confidence Interval; ESCC, esophageal squamous cell carcinoma; HR: hazard ratio; YTHDF1: YTH N6-methyladenosine RNA-binding protein 1; HNRNPC: heterogeneous nuclear ribonucleoprotein C; * Meaningful *p*-value.

## 4 Discussion

ESCC is a highly malignant tumor, and in the pathogenesis of ESCC, genetic and epigenetic modifications play a key role in the occurrence and development of ESCC (Cheung and Liu, 2009; Fatehi Hassanabad et al., 2020). At present, surgery combined with radiotherapy, chemotherapy and targeted therapy is the main treatment method for ESCC. However, its high recurrence and metastasis rates make the survival outcome of ESCC unsatisfactory (Siegel et al., 2019; Chen et al., 2020). Therefore, there is an urgent need to find new diagnostic biomarkers and potential therapeutic targets for ESCC patients. m6A methylation is the most common form of mRNA modification and has been shown to play an important role in the formation of a variety of tumors (Su et al., 2019), and the discovery of m6A opens up a new way for the study of epigenetics and tumor-related diseases. Previous literature reported that the expression levels of intracellular “writer” and “eraser” genes determined the methylation level of m6A, while the protein expressed by the “reader” gene can bind to m6A methylation sites to execute biological functions (Sun et al., 2019). At present, the study of m6A in ESCC is still in its infancy, but in other tumors, m6A-related genes have been confirmed as potential tumor diagnostic markers. Therefore, this study used machine learning methods to find out ESCC-related m6A regulators, providing new targets for the development of clinical molecular targeted therapeutic drugs in the future.

In this study, we first analyzed the expression of 21 m6A regulators that were significantly different between ESCC and normal tissues using RNA-seq transcriptional data from 66 pairs of ESCC and normal tissues from our hospital. The expression profiles of 11 m6A methylation regulators were also analyzed using RNA-seq transcriptional data from the TCGA-ESCA database. Subsequently, we assessed the m6A regulators most significantly associated with ESCC occurrence by means of machine learning. Before applying machine learning for analysis, the best machine learning model should be identified firstly. In terms of our hospital RNA-seq data, we applied RF and SVM models to evaluate the accuracy of model predictions. Through boxplots and cumulative residual distributions, we determined that the RF model’s predicted values differed less from the true values and the model was more accurate. Therefore, we chose the RF model to predict ESCC-related m6A genes. Finally, we screened out IGFBP3, HNRNPA2B1, YTHDF1, YTHDC1, HNRNPC, YTHDF3, WTAP, FTO and RBM15 as m6A regulators significantly associated with ESCC according to the meanDecreaseGini score. We then used the same approach to model the RNA-seq transcription data of TCGA-ESCA, and also found that the RF model was more accurate for prediction. HNRNPC, FMR1, YTHDF1 were confirmed as the m6A regulators significantly associated with ESCC in the RNA-seq transcription data of TCGA-ESCA by meanDecreaseGini score. After overlapping the machine learning results of the two databases, we generated a two-gene risk assessment model consisting of YTHDF1 and HNRNPC, and showed good performance in predicting the occurrence of ESCC. Finally, we analyzed the relationship between the two-gene risk assessment model of YTHDF1 and HNRNPC and clinicopathological features, and validated the expression of YTHDF1 and HNRNPC in clinical tissues by WB and IHC staining. The prognostic significance of YTHDF1 and HNRNPC were also identified through IHC staining, which not only indicated that YTHDF1 and HNRNPC were involved in the development of ESCC, but also affected the prognosis of ESCC patients.

YTH N6-methyladenosine RNA-binding protein 1 (YTHDF1) belongs to the YTH domain family and is a “reader” for m6A-modified mRNA. Recent studies have shown that YTHDF1 is involved in the occurrence and development of various cancers. YTHDF1 promotes lung cancer progression through its involvement in the m6A demethylase ALKBH5 pathway (Jin et al., 2020b). In bladder cancer, YTHDF1 promotes bladder cancer growth and progression through the ITGA6-METTL3 pathway (Jin et al., 2019). In colorectal cancer, YTHDF1 plays an important oncogenic role in cellular self-renewal and differentiation through the Wnt/β-catenin pathway (Bai et al., 2019). In esophageal cancer, only Wang et al. reported that HCP5 can directly interact with YTHDF1 to promote the binding of YTHDF1 to m6A-modified HK2 mRNA, enhancing the stability of HK2, thereby promoting the progression of ESCC (Wang et al., 2022). In our study, YTHDF1 was highly expressed in ESCA tissues, and higher expression of YTHDF1 was associated with poorer survival, suggesting that YTHDF1 may act as a tumor-promoting gene in ESCC, but the specific tumor-promoting mechanism still needs further study.

HNRNPC, an RNA-binding protein, is a member of the heterogeneous ribonucleoproteins (hnRNPs). HNRNPC contains only one RNA-recognition motif (RRM), which must interact with its RNA target after oligomerization into tetramers (Cieniková et al., 2015). It can selectively recognize m6A mRNA sites that mediate mRNA degradation (Berlivet et al., 2019), and it has also been identified as a “reader” for m6A modifications (Nettersheim et al., 2019). HNRNPC can regulate non-specific RNA export, RNA expression, stability, and 3′-end processing and translation of RNA splicing sequences (Wu et al., 2018). It plays an important role in the occurrence and development of various cancers. Huang et al. (Huang et al., 2020) reported that HNRNPC was overexpressed in oral squamous cell carcinoma (OSCC), and higher HNRNPC expression levels were positively associated with poor overall survival. Meanwhile, HNRNPC was shown to promote the proliferation, migration and invasion of OSCC cell lines. Wu et al. (Wu et al., 2018) and Huang et al. (Huang et al., 2016) reported that higher expression of HNRNPC was significantly associated with worse OS in both breast and gastric cancers. In esophageal cancer, there are few studies on HNRNPC. Among them, Xu et al.'s bioinformatics analysis combined with ESCC independent cohort studies found that higher expression of HNRNPC in ESCC tissue was associated with poorer prognosis of patients, furthermore, HNRNPC and ALKBH5 have been shown to be prognostic indicators associated with ESCC survival outcomes (Xu et al., 2020). In this study, HNRNPC was confirmed to be significantly related to the pathogenesis of ESCC, and HNRNPC was highly expressed in ESCC tissues. At the same time, IHC confirmed that HNRNPC was an independent prognostic factor of ESCC, which was consistent with the previous studies’ results and further confirmed that HNRNPC promotes ESCC progression. However, both YTHDF1 and HNRNPC act as m6A readers, and they need to identify methylated genes or m6A writers to promote tumor development, therefore, we next performed a PPI analysis of both YTHDF1 and HNRNPC in the transcriptome data of our hospital, respectively, where the connection between HNRNPC and METTL3 has been constructed. Methyltransferase-like protein 3 (METTL3) is the most important component of the m6A MTC and is highly conserved in eukaryotes from yeast to humans (Chen Z et al., 2021). METTL3 has also been confrimed to be highly expressed in ESCC and is associated with poor prognosis in esophageal cancer (Xia et al., 2020). At the same time, regarding the relationship between ESCC and YTHDF1, Lin et al. once reported that METTL3 induced epithelial-mesenchymal transition (EMT), which was an early metastasis event of YTHDF1-mediated m6A-increased Snail mRNA translation (Lin et al., 2019). Our study established the association between METTL3 and HNRNPC as well, meanwhile, our group has also been studying the specific mechanism of m6A lied in the progression of ESCC, we hope the current results will make contribution to all the scholars for future in-depth research.

The advantages of our research lie in the following aspects: 1) We used large sample RNA-seq data to find ESCC-related m6A regulators, and used the TCGA database for verification. We then performed further analysis on the overlapped genes to ensure the reliability and authenticity of our results. 2) For the above searched ESCC-related m6A regulators, we performed WB experiments in clinical tissues, and validated the results of bioinformatics analysis with ESCC independent cohorts using IHC methods. The experimental results are consistent with the results predicted by bioinformatics. 3) This study is different from previous studies because we used machine learning methods to find m6A regulators related to ESCC occurrence. Machine learning has been widely used in the diagnosis, classification and prognosis of ESCC, and these studies have shown that machine learning is more accurate than logistic regression (Chen X et al., 2021; Li et al., 2021; Zhang et al., 2022). In clinical statistics, the traditional classification model is Logistic regression analysis. The disadvantage of this model is that when the feature space is large, the performance of logistic regression is not very good. Meanwhile, it is prone to underfitting, the accuracy is not very high, and it is unable to handle a large number of multi-class features or variables well. Based on these, more and more machine learning methods are used in the field of medical statistics (Watson et al., 2019), (Darst et al., 2018). In this study, we also directly chose machine learning instead of logistic regression analysis as the main method to screen ESCC-related m6A regulators. After reviewing the machine learning studies related to ESCC, there are no studies using machine learning to screen ESCC-related m6A regulators. In addition, this study performed a visual analysis of the nomogram of the model, and further used calibration curves, clinical decision curves, and clinical impact curves to evaluate the predictive ability of the model and the ability to maximize its benefits, which all showed that the model has a good classification advantage.

## 7 Conclusion

In this study, we used transcriptome data of our hospital and TCGA-ESCC database to screen out two ESCC-related m6A RNA methylation regulators through machine learning: YTHDF1 and HNRNPC. The association of the expression of YTHDF1 and HNRNPC with the prognosis and clinicopathological characteristics of ESCC patients was verified by WB and IHC. Based on our constructed risk prediction model of m6A RNA methylation regulators, this may provide important information for developing diagnostic and therapeutic strategies in the future.

## Data Availability

The datasets presented in this study can be found in online repositories. The names of the repository/repositories and accession number(s) can be found in the article/Supplementary Material.

## Abbreviations

AUC, Area Under Curve; CIC, Clinical Impact Curves; EC, Esophageal cancer; ESCC/ESCA, Esophageal quamous cell carcinoma; HR, Hazard Ratio; HRP, Horseradish Peroxidase; HNRNPC, RNA-binding protein, is a member of the heterogeneous ribonucleoproteins C; IGF2BP1-3, Insulin-like growth factor 2 mRNA-binding proteins 1-3; m6A, N6-methyladenosine; METTL3, Methyltransferase-like 3; METTL14, Methyltransferase-like 14; NCCR, National Central Cancer Registry of China; OS, Overall Survival; OSCC, oral squamous cell carcinoma; PBS, Phosphate Buffer Saline; RF, Random Forest; RRM, RNA-recognition motif; SVM, Support Vector Machine; TCGA, The Cancer Genome Atlas; YTHDF1, YTH N6-methyladenosine RNA-binding protein 1; YTHDC1-2, YTH domain-containing 1-2.
